# Impact of Renal Replacement Therapy on Mortality in Critically Ill Patients—The Nephrologist’s View within an Interdisciplinary Intensive Care Team

**DOI:** 10.3390/jcm10153379

**Published:** 2021-07-30

**Authors:** Matthias Klingele, Lea Baerens

**Affiliations:** 1 Department of Internal Medicine, Nephrology and Hypertension, Saarland University Medical Centre, 66424 Homburg/Saar, Germany; lea.baerens@web.de; 2 Department of Nephrology, Hochtaunuskliniken, 61352 Bad Homburg, Germany

**Keywords:** dialysis, acute kidney injury, mortality

## Abstract

Acute kidney injury (AKI) is a common complication in critically ill patients with an incidence of up to 50% in intensive care patients. The mortality of patients with AKI requiring dialysis in the intensive care unit is up to 50%, especially in the context of sepsis. Different approaches have been undertaken to reduce this high mortality by changing modalities and techniques of renal replacement therapy: an early versus a late start of dialysis, high versus low dialysate flows, intermittent versus continuous dialysis, anticoagulation with citrate or heparin, the use of adsorber or special filters in case of sepsis. Although in smaller studies some of these approaches seemed to have a positive impact on the reduction of mortality, in larger studies these effects could not been reproduced. This raises the question of whether there exists any impact of renal replacement therapy on mortality in critically ill patients—beyond an undeniable impact on uremia, hyperkalemia and/or hypervolemia. Indeed, this is one of the essential challenges of a nephrologist within an interdisciplinary intensive care team: according to the individual situation of a critically ill patient the main indication of dialysis has to be identified and all parameters of dialysis have to be individually chosen with respect to the patient’s situation and targeting the main dialysis indication. Such an interdisciplinary and individual approach would probably be able to reduce mortality in critically ill patients with dialysis requiring AKI.

## 1. History of Dialysis

Acute kidney injury (AKI) is a common complication in critically ill patients. Severe AKI leads to three classical life-threatening complications which may occur alone or in combination: hypervolemia, hyperkalemia and uremia. Without renal recovery, these complications historically resulted in 100% mortality. The first successful dialysis was conducted by Kolff in 1945 with survival of the patient suffering from life threatening severe AKI. Since then, dialysis became a common treatment of the major complications of AKI, hypervolemia, hyperkalemia and uremia, resulting in reduced mortality.

**Figure 1:**
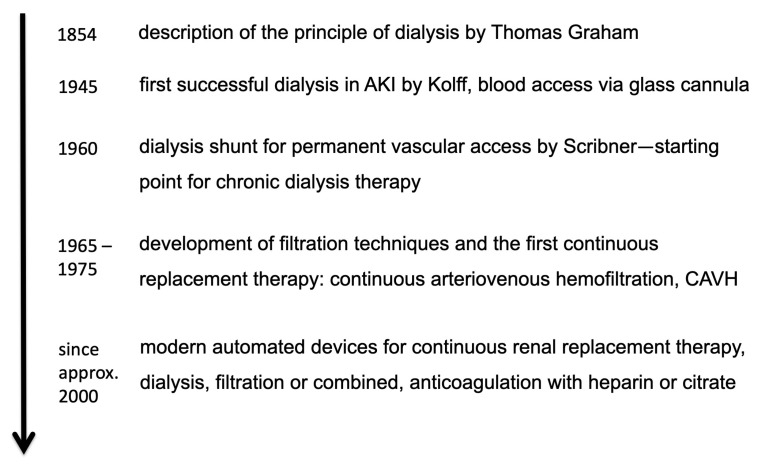
History of dialysis and development of renal replacement therapy.

Since 1945 dialysis has continuously been improved and treatment options have been broadened as shown in Figure 1:

- In the beginning, dialysis was considered as a short-term treatment in the case of severe AKI. Later, dialysis became an option for long-term treatment in the case of persistent kidney failure. Today, chronic dialysis is an established long-term therapy option.
- Nowadays, dialysis machines have been developed up to computer-controlled high-tech machines. These modern dialysis machines with several automatic monitoring and control functions ensure a safe and efficient dialysis treatment. Therefore, kidney replacement therapy can be carried out in practically any intensive care unit.
- Compared to the first dialysis, nowadays a variety of therapy options for different purposes are available, different choices of dialysis or filtration techniques can be chosen combined with a high variability of dialysate composition and flow rates.

Despite all the technical advances, the most common indications for dialysis have remained the same since 1945: hypervolemia, hyperkalemia and/or uremia—the major complications of acute kidney injury.

## 2. AKI and Dialysis in Critically Ill Patients

AKI is defined as an increase in serum creatinine, a decrease in the amount of urine, or both. The severity of an AKI is classified according to the AKIN criteria [1]. Around 10%–15% of hospitalized patients suffer from AKI; the incidence is up to 50% in intensive care patients [2]. The mortality of patients with AKI requiring dialysis in the intensive care unit is up to 50%, especially in the context of sepsis [3].

There have been various considerations and approaches of how to reduce high mortality in critically ill patients with kidney failure requiring dialysis which are briefly discussed below. To facilitate understanding, the discussion focuses on continuous renal replacement therapy (CRRT) as it is mostly used in critically ill patients. Different techniques are available for CRRT, filtration (CVVH) or dialysis (CVVHD). In the following, the term “CRRT” refers to CVVHD. Any given statement further also applies analogously to CVVH, meaning here applied to the substitute instead of the diaylsate.

- **When to start dialysis?**

Dialysis should be started at the latest when the complications of an AKI can no longer be controlled under conservative therapy. In contrast, an early start of renal replacement therapy is based on the idea of not exposing the organism to the complications of AKI, hoping to thereby influence mortality. However, the results of studies analyzing early versus late start of dialysis are quite heterogeneous with regard to mortality [4,5,6]. Taken together, a reduction in mortality may exist for certain situations, such as an AKI in the context of an elective surgical procedure [6]. However, in most studies an early start of dialysis had no beneficial influence on mortality, especially not in patients with sepsis and/or multiple organ failure [4,5].

- **Which dialysis dose?**

Another aspect to reduce mortality was the attempt to apply the most effective dialysis possible, thus enhancing the dose in CRRT. In clinical practice the dialysis dose depends mostly on the volume of dialysate per hour (h) with respect to the patient’s body weight and is defined as dialysate flow in ml per kg body weight and h. Although the first studies showed promising results [7], an effective reduction in mortality by increasing the dialysate flow to more than 30 mL/kg body weight/hour could not be proven in the following [8,9]. However, there is consensus that dialysate flow should reach at least 25 mL per kilo of body weight per hour in order to ensure effective dialysis or adequate dialysis quality.

- **Which modality of dialysis?**

The choice of an intermittent dialysis (IHD) or continuous renal replacement therapy (CRRT) is closely linked to circulatory stability and volume balance. Hypervolemia in critically ill patients with AKI is a prognostically unfavorable parameter. Therefore, studies were undertaken to correct the volume balance at an early stage with the help of dialysis, if necessary, even before a “hard” dialysis indication had been established formally [10]. Controlling fluid balance and avoiding volume overload with CRRT seems to be more favorable for the critically ill. However, the effects of the dialysis modality (intermittent or continuous) are not significant enough to sustainably improve mortality or to derive clear recommendations [11]. Nevertheless, there is consensus that CRRT allows volume correction more easily while additionally providing better circulatory stability compared to IHD [12]. Moreover, studies have shown that CRRT may have a positive impact on renal recovery compared to IHD [13].

- **Which anticoagulation?**

In order to perform renal replacement therapy adequate anticoagulation is necessary. Heparin for anticoagulation is historically closely linked to the success of dialysis in 1945 and is still often used [14]. Anticoagulation with heparin is targeted on the technical needs of effective dialysis. This increases the risk of bleeding in critically ill patients, especially in the context of coagulation disorders or surgery interventions. Regional anticoagulation with citrate was developed around 40 years ago [15] and enables an effective extracorporeal anticoagulation without impairing or influencing the patient’s systemic coagulation and lowers the risk of bleeding complications [16] compared to heparin [17]. Some studies reported that citrate anticoagulation could possibly have a positive influence on mortality in sepsis and multiple organ failure compared to heparin [14], based on the idea that citrate could reduce calcium-dependent inflammatory processes with a positive influence on the outcome. However, this was not proven in larger studies [18]. Relevant side effects of citrate-based regional anticoagulation can be alkalosis [19] or citrate accumulation with accompanying hypercalcemia. However, such complications seem to depend on the level of experience in proper handling of citrate anticoagulation [20]. In addition, it has been shown that fears of inadequate citrate metabolism due to impaired liver function are groundless in situations where appropriate caution is taken [21].

- **Special filters and adsorbers?**

Bacterial toxins and an excessive cytokine response are the main causes of the development of sepsis or are responsible for their effects in the body, including the development of organ failure. Adsorbers have been developed to adsorb cytokines and/or toxins in parallel with renal replacement therapy in order to address sepsis and the cytokine storm in addition to the therapy of AKI [22]. Furthermore, very high dialysate flows and different filtration techniques or special filters were used in some cases. In smaller studies, such procedures sometimes showed a positive effect on the outcome. Although all these techniques are able to reduce cytokines or bind bacterial toxins, the promising results of smaller studies regarding outcome have mostly not yet been confirmed in larger studies [23,24].

- **Special extracorporeal elimination procedures**

Extracorporeal therapy is not limited to different modalities of dialysis, filtration or the use of special filters, adsorbers, etc. Moreover, in critically ill patients the elimination of every molecule/toxin, etc., can be of clinical interest. This is one of the essential challenges of a nephrologist within an interdisciplinary team to realize in the case of needing to eliminate every kind of substance from the blood/the patient. For this, plasmapheresis and liver replacement therapy are essential and indispensable methods—even though not often used compared to dialysis. Mostly, these methods are used in specialized centers with interdisciplinary intensive care units. Thus, for a nephrologist, the central question is, what should be eliminated from the blood—independently of the physico-chemical aspect of the target molecule, which determines the method to use (water soluble = dialysis or filtration).

The idea of removing pathological substances from the blood that cannot be eliminated by dialysis is the basis of extracorporeal procedures such as liver replacement therapy or plasmapheresis. The challenge of liver failure lies in the presence protein-bound substances that cannot be eliminated by dialysis. Instead, liver replacement therapy has been developed. However, these systems are quite complex—regardless of the technology used. Although these systems had been developed further, liver replacement therapies have not significantly reduced mortality from liver failure compared to a conservative treatment approach [25]. In view of the high costs and the very high expenditure of work for liver replacement therapy, and that only a few employees in a center can handle the complex technology, liver replacement therapies are rarely used outside specialized centers. Plasmapheresis also does not play a major role in everyday clinical practice and is only used in exceptional cases in the critically ill—with the exception of neurological indications such as severe myasthenia or Guillain Barré syndrome requiring intensive care [26].

## 3. The Dilemma: Despite RRT in the Critically Ill with AKI Persistent High Mortality

With this in mind, why is it that modern dialyses with a high dialysate flow, an early start of CRRT or anticoagulation with citrate, but also specific procedures such as liver replacement procedures, pursue an impressively logical approach of how substances in the blood can be eliminated, but do not seem to have a positive effect on mortality?

Renal replacement therapy was developed to delay acute death from complications of AKI—at least for a certain time. Since the first successful dialysis in 1945, hardly anything has changed in terms of the objectives of renal replacement therapy. It is true that the further development of dialysis machines and materials led to a significant reduction in mortality from the complications associated with the dialysis itself. 

However, today the demands made on renal replacement therapy go well beyond the goal to control the complications of kidney failure without causing complications. At the same time, renal replacement therapy may also reduce global mortality. For this, start of dialysis, dialysate flow or certain filters and filtration techniques have been changed over time to hopefully reduce global mortality, but unfortunately without reaching this goal, especially in the case of sepsis and/or multiple organ failure.

**Figure 2:**
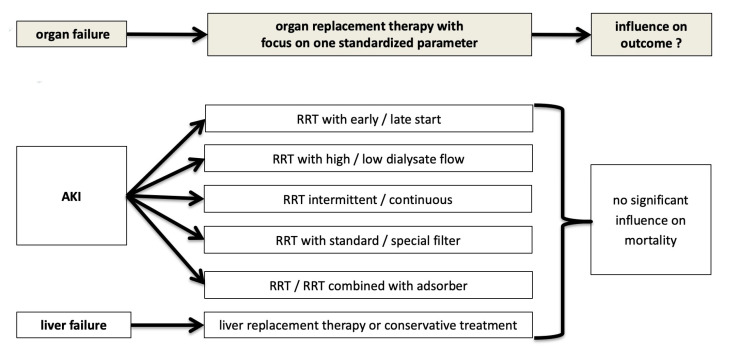
Classic approach in large studies on AKI and RRT in critically ill patients: selecting a single variable as target.

This phenomenon, that in larger clinical trials convincing treatment options did not really have a beneficial impact on outcome in septic patients, has also been described independently of AKI and RRT [27,28]. An increasingly shared concept is that treatment option in critically ill patients can only result in a benefit if the right patient is chosen for an appropriate indication and appropriate treatment [29]. This may explain negative trials in heterogeneous patient populations when a single variable was selected for evaluation, as shown in Figure 2 since some patients may benefit and some may be harmed in both treatment groups with an overall globally negative result [29].

## 4. AKI and Dialysis in the Context of Multiple Organ Failure: The Nephrologist’s Attempt at Explanation

In order to find an explanation to why mortality in patients with AKI requiring RRT may be still quite high, sepsis with multiple organ failure shall be illustrated within a metaphor. Imagine a tree: the trunk is the sepsis, the individual organ failure (kidney, liver, circulation, respiration, etc.) constitutes the larger branches. Many above-mentioned studies were investigating whether trimming a tree with scissors from the left or the right side would have an influence on the survival of the whole tree in the case of a severely ill trunk. It is clear to everyone: whether the branch of a tree with ill trunk is “treated” with scissors from the left or the right side will have little or even no influence on the survival of the entire tree, as long as the action carried out neither leads to healing the trunk nor vice versa, damaging the tree further.

Applied to renal replacement therapy and its influence on the mortality of critically ill patients, this means: a bleeding complication, e.g., under heparin, can negatively affect mortality. In contrast, the influence of renal replacement therapy on the outcome seems to be rather small, apart from life threatening complications of AKI. 

Therefore, has kidney replacement therapy really exerted any influence on the underlying disease or its course and, thus, the outcome? Looking again at the picture of the tree: if a branch of a tree suffers from a disease, it is ideally treated as specifically as possible. Lice are treated in a different manner than a fungal attack—both diseases ultimately have different effects on the entire tree.

**Figure 3:**
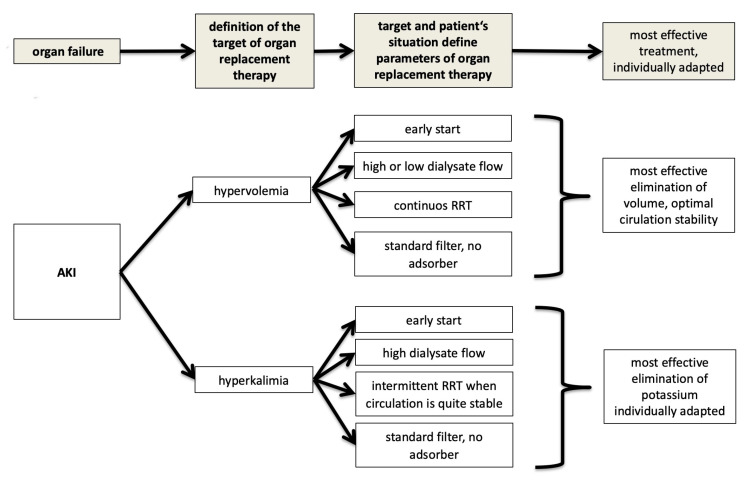
Individualized approach for RRT.

Looking at AKI, this means that dialysis should be directed towards the leading complication of kidney failure with focus on its treatment, as shown in Figure 3. 

Consequently, besides avoiding death from complications of kidney failure, such a focused dialysis has the greatest benefit for the rest of the body as well. For example, in the case of hypervolemia kidney replacement therapy should be targeted accordingly. Thus, ultrafiltration is of central importance, since hypervolemia threatens the entire organism. At the same time, ultrafiltration can negatively influence the patient’s circulation. Therefore, CRRT would be advantageous. In contrast, parameters such as the dialysate flow or the choice of the filter have no influence on hypervolemia and, therefore, will not have any impact on the patient’s survival.

Another example to explain why in different situations different parameters of renal replacement therapy can have an influence on survival or not is hyperkalemia: in this indication ultrafiltration has no impact on elimination of potassium. Instead, dialysate flow is of central interest, since the higher the dialysate flow, the more potassium can be eliminated over time. In the case of a patient having stable circulation, an IHD can even be more advantageous compared to CRRT, due to the possible high dialysate volumes in IHD.

These two examples demonstrate that renal replacement therapy can only be effective if all parameters relevant for dialysis are adjusted to the target, the central indication of dialysis. With such an individually customized approach a positive influence of renal replacement therapy on the outcome could be conceivable. This has been demonstrated in a well selected population, such as burn patients with severe septic shock and AKI: the use of plasma sorbent technology has shown a positive impact on mortality compared to standard CRRT treatment [30].

Unfortunately, the majority of studies were carried out on the basis of a heterogeneous patient population, with different indications for dialysis and also heterogeneous dialysis parameters. This may explain why these studies have not shown any significant influence of renal replacement therapy on outcome in critically ill patients with AKI, especially when comparing a single parameter, e.g., early versus late start of dialysis or high versus low dialysate flow, comparable to what has been described by Vincent [29]. 

Therefore, the expected positive influence of an individually tailored renal replacement therapy on outcome—from a nephrological point of view—is only possible by an individually customized, case by case approach. The first step then focuses on why, or with what target, dialysis should be started. The second step regards the demands on the parameters of dialysis, e.g., dialysate flow or ultrafiltration, which are essential to actually reach the initially defined target by dialysis. Thirdly, the defined parameters have to be individually tailored to the patient, maybe even adjusted over time according to the patient’s progress. This individualized approach reflects the role of a nephrologist in a multidisciplinary team related to their competence in the management of AKI including extracorporeal treatment options [31]. Moreover, a nephrologist provides experience for critically ill patients with complex diagnoses and AKI and ideally supports the education in this field to help to assess quality and improve care [32].

## 5. Conclusions

What is the conclusion of a nephrologist when being asked about renal and, in rare cases, liver replacement therapy in critically ill patients?

Each patient should be considered individually: the underlying disease must be clearly worked out, then the goal of organ replacement therapy should be defined within an interdisciplinary dialogue, e.g., hypervolemia or hyperkalemia. This approach automatically answers the question of an early or rather late start of dialysis. Moreover, parameters of dialysis, e.g., the dialysate flow rates, are individually defined in order to achieve the target: dialysis dose should be at least 25 mL dialysate/kg body weight/hour; in the case of hyperkalemia or acidosis, however, significantly higher dialysate flows must be selected in order to achieve adequate elimination. The use of CRRT and citrate anticoagulation is actually standard in many hospitals and suggested by current guidelines with regard to circulatory stability and bleeding complications. Special filters and/or adsorbers should only be chosen in clearly defined situations. In the case of usage, it is essential to match the settings such as dialysate and blood flow to the special filters used and objectives defined, for example, choosing a filter for the elimination of myoglobin in severe rhabdomyolysis requires high flow rates. Otherwise, such a special filter does not provide any measurable value added compared to “standard dialysis”. Therefore, specific filters and adsorbers should only be used in centers that have the appropriate experience.

Ideally, there is very close and trusting cooperation between the intensive care physicians and the nephrologist in a center. As a result, all aspects relevant to an organ replacement procedure are worked out together through an individual plan for each patient. If necessary, this will be adapted to changing circumstances at any time.

The author’s personal experience allows the conclusion that an interdisciplinary team working fully together has a positive impact on the mortality of critically ill patients with dialysis requiring AKI.
